# Arithmetic and Biologically-Inspired Computing Using Phase-Change Materials

**DOI:** 10.1002/adma.201101060

**Published:** 2011-06-22

**Authors:** C David Wright, Yanwei Liu, Krisztian I Kohary, Mustafa M Aziz, Robert J Hicken

**Affiliations:** School of Engineering, Computing and Mathematics, University of ExeterExeter EX4 4QF, UK E-mail: david.wright@exeter.ac.uk; School of Physics, University of ExeterEX4 4QF, UK

**Keywords:** phase-change materials, phase-change memories, memristors, non von-Neumann computing

Computers in which processing and memory functions are performed simultaneously and at the same location have long been a scientific “dream”, since they promise dramatic improvements in performance along with the opportunity to design and build ‘brain-like’ systems.^1^–^3^ This “dream” has moved a step closer following recent investigations of so-called memristor (memory resistor) devices.^4^–^8^ However, phase-change materials also offer a promising route to the practical realisation of new forms of general-purpose and biologically-inspired computing.^9^–^11^ Here we provide, for the first time, an experimental proof-of-principle of such a phase-change material-based “processor”. We demonstrate reliable experimental execution of the four basic arithmetic processes of addition, multiplication, division and subtraction, with simultaneous storage of the result. This arithmetic functionality is possible because phase-change materials exhibit a natural accumulation property, a property that can also be exploited to implement an “integrate and fire” neuron.^12^, ^13^ The ability of phase-change devices to ‘remember’ previous excitations also imbues them with memristor-type functionality,^4^, ^8^ meaning that they can also provide synaptic-like learning.^6^, ^7^, ^13^ Our results demonstrate convincingly these remarkable computing capabilities of phase-change materials. Our experiments are performed in the optical domain, but equivalent processing capabilities are also inherent to electrical phase-change devices.

Phase-change materials such as GeSbTe or AgInSbTe alloys exhibit some remarkable properties; they can be crystallised by pulses in the picosecond range,^14^, ^15^ yet can remain stable against spontaneous crystallisation for many years. They show hugely contrasting properties between amorphous and crystal phases, including an electrical conductivity difference of up to five orders of magnitude^16^ and a large refractive index change; properties that have led to their application in electrical (phase-change RAM or PCM devices) and optical (DVD and Blu-Ray discs) memories.^17^, ^18^ The origin of such remarkable properties has been a source of much recent research. Kolobov^19^ showed that, contrary to expectations, the short-range order in GeSbTe is higher in the amorphous than in the crystal phase. This was explained by an ‘umbrella flip’ of Ge atoms, which was put forward as the potential origin of ultra-fast switching. The crystalline phase of phase-change alloys is also unusual, exhibiting strong resonance bonding, with such bonding being suggested as a ‘necessary condition’ for technologically useful phase-change properties.^20^ The scientific and technological importance of phase-change materials is clearly high; however their use for simple binary storage, the main application to date, barely begins to exploit their remarkable properties to the full. As pointed out by Ovshinsky,^9^, ^10^ some phase-change materials, such as GeSbTe, should also be capable of non-binary arithmetic processing, multi-value logic and biological (neuromorphic) type processing. The origins of these exciting possibilities lie in the detail of the crystallisation process in nucleation- dominant materials.^11^

Crystallisation can be viewed as energy-accumulation, with excitation “events” (electrical or optical pulses) as the energy source. For binary storage the aim is to ensure complete crystallisation with a single excitation. For phase-change based processing however, multiple excitations that exploit the natural accumulation property are used. For example, in conventional (electrical) PCM devices we can control excitation voltage and current such that only a partial crystallisation occurs with each excitation.^21^ With a succession of such excitations, nano- crystallites are formed which may grow and merge to form conducting pathways, at which point the cell resistance changes quite abruptly (see **Figure** **1**a). Analogous behaviour occurs using optical excitation (the experimental method we use here), and can be understood using a physically realistic crystallisation model. One such model is the rate-equation approach^11^, ^22^ that tracks both sub-critical and super-critical crystal cluster sizes during each excitation event. The ability to track sub-critical clusters is important since they play a significant role in the early stages of crystallisation, as recently confirmed experimentally.^23^ Our rate-equation model is discussed in detail elsewhere^11^, ^22^ (Supporting Information); here we use it to understand the processing capability of the energy accumulation regime. For this we consider a region of phase-change material, here the nucleation-dominant material Ge_2_Sb_2_Te_5_, subject to a series of optical or electrical excitations. For simplicity we assume that as a result of each excitation the entire region is heated to some constant temperature *T_excite_* for a duration *Δt* seconds. We calculate the population distributions of crystal cluster sizes before, during and after each excitation and track the fraction of crystallised material. We map the change in crystal fraction to a change in electrical and optical properties using effective medium theory^24^, ^25^ (Supporting Information). In Figure 1b we show the calculated optical reflectivity and electrical conductivity as a function of the number of excitations assuming a fully amorphous starting phase, T_excite_ = 700 K (chosen to match the estimated temperature achieved in our experimental results–see Supporting Information) and various pulse durations; initially there is relatively little change in optical reflectivity or electrical conductivity but a distinct threshold exists where a rapid change sets in, with the suddenness of the change in electrical properties being more pronounced (due to percolation). The number of pulses required to reach the threshold can be controlled via the excitation duration (or amplitude). In this example we have applied excitations sequentially (a format suited to arithmetic processing); however, for multiple weighted parallel inputs, as shown schematically in Figure 1a, we can use the same accumulation, threshold and non-linear output change (in resistance or reflectivity) to mimic an ‘integrate and fire’ biological neuron^12^, ^13^ using a single phase-change cell (or spot), a far simpler approach than conventional implementations that use relatively complicated multi-transistor CMOS circuits^26^ (although we note that similarly simple neuron-like hardware can be implemented using non-phase-change based memristive systems^27^, ^28^)

**Figure 1:**
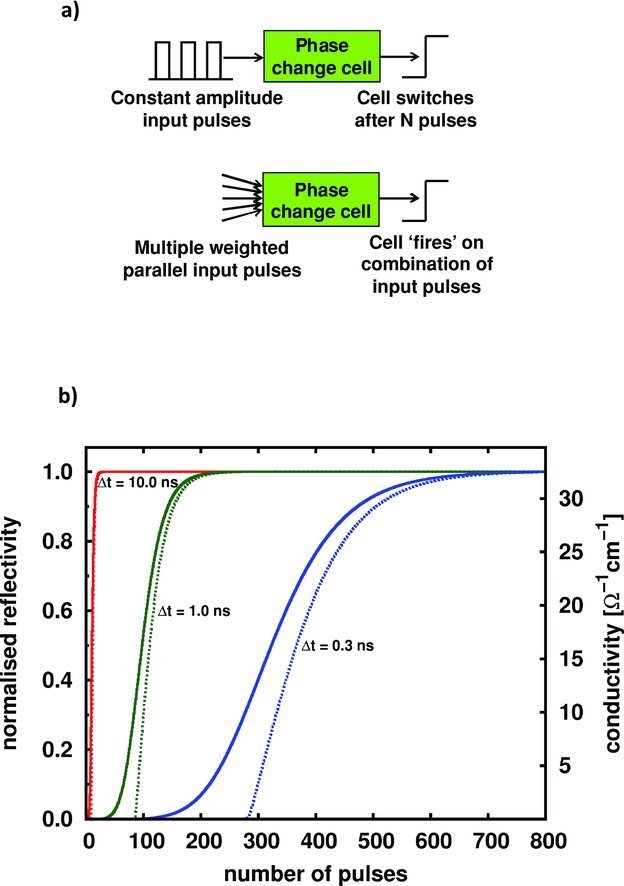
Processing using the accumulation property of GeSbTe. a) Schematic of phase-change processor for arithmetic (top) and neuron-like (bottom) processing. b) Simulated, using the rate-equation and effective medium theories, change in normalized reflectivity (solid lines) in a Ge_2_Sb_2_Te_5_ sample as a function of the number of 700 K temperature excitations (rectangular temperature pulses) of duration 10 ns, 1 ns, and 0.3 ns. Also shown is the resulting change in sample conductivity (dashed line). The natural accumulation and threshold property of phase-change materials is clear.

We now implement experimentally a phase-change arithmetic processor, working in the optical regime. The optical arrangement is shown in **Figure** **2**a and comprises a pulsed pump beam and a continuous probe beam that are overlapped on the sample surface within the focal plane of an optical microscope. The pulsed beam excites the phase-change material (here a Si/ZnS-SiO_2_(310 nm)/Ge_2_Sb_2_Te_5_ (20 nm)/ZnS-SiO_2_ (30 nm) sample typical of that used in optical storage discs) while the probe beam measures the reflectivity. We used 800 nm pump pulses in the range 70 fs to 500 fs and fluences from 2 mJ cm^−2^ to 12 mJ cm^−2^. The typical reflectance change as a function of the number of pulses is shown Figure 2b, for which case the sample remains in the accumulation mode with little or no change in reflectivity until around 150 pulses are received, whereupon subsequent pulses cause significant increases in reflectivity. In this arrangement the system might be used to perform arithmetic computations in a high-order base. More usefully, individual pulses can be combined into groups with each group designating a single excitation event. This approach gives great flexibility; for example if a single excitation comprises 25 successive 85 fs, 3.61 mJ cm^−2^ pulses of the form used in Figure 2b, then a threshold between the 9^th^ and 10^th^ excitation can be readily set (suitable for base-10 addition and multiplication). Combining the same individual pulses into groups of 16 would on the other hand provide a threshold suitable for direct hexadecimal computations. The response curve for our base-10 scheme is thus as shown in Figure 2c; note that there is very little reflectance change for the first 6 to 7 excitations, and that the change for 10 excitations (6%) is significantly larger than that for 9 excitations (4%) and a suitable reflectivity threshold for computations is 5% in this case. Also shown in Figures 2b and 2c for comparison is the simulated, using the rate-equation model and effective medium theory, change in reflectivity; to evaluate the theoretical results we calculated the temperature distribution in the Ge_2_Sb_2_Te_5_ sample by analytical solution of the heat equation for an impulsive optical source (we note that our analytical thermal model does not include phonon-carrier interaction and relaxation processes often included in more complex two-temperature type models^29^ of fast thermal processes; however the crystallisation process will be dominated by the relatively long (ns order) thermal time constant of the optical disc-like sample used here, rather than the very short thermalisation time which is typically less than 5 ps for Ge_2_Sb_2_Te_5_^30^ – see Supporting Information for more details).

**Figure 2:**
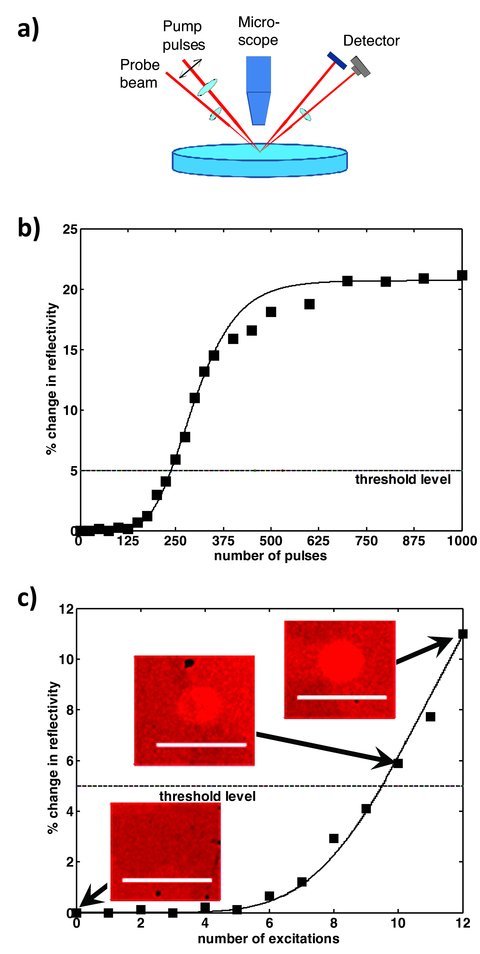
Experimentally measured accumulation property of Ge_2_Sb_2_Te_5_. a) Schematic of the set up for the femtosecond laser experiments. b) Experimentally measured (squares) change in optical reflectivity ((R–R_a_)/R_a_) where R_a_ is amorphous phase reflectivity) of the Ge_2_Sb_2_Te_5_ sample as a function of the number of 85 fs, 3.61 mJ/cm^2^ pulses applied. c) Experimentally measured (squares) change in reflectivity as a function of excitation events (for first 12 events), with a single excitation event comprising 25 × 85 fs, 3.61 mJ/cm^2^ pulses and chosen so that a threshold can be set for the implementation of base-10 addition and multiplication. Result shows clearly the energy accumulation property and the threshold (at 5% change in optical reflectivity) is set between the 9^th^ and 10^th^ excitations; also shown are microscopic images of the mark formed after 10 excitations (6.3% change in reflectivity) and after 12 excitations (11% change in reflectivity), as well as the initial amorphous starting phase (white scale bar is 50 μm). Also shown in 2b and 2c is the simulated change in reflectivity (solid lines), calculated using the rate equation and effective medium models and a sample temperature distribution obtained by analytical solution of the heat conduction equation for an impulsive optical source (Supporting Information).

Now we are ready to implement base-10 addition. Having already set the threshold change in optical reflectivity to occur between the 9^th^ and 10^th^ excitations as in Figure 2c, we can compute a base-10 addition directly by inputting a number of excitations equal to the first addend, followed by excitations equal in number to the second addend.^9^, ^11^ The phase-change ‘processor’ automatically sums the two addends due to its accumulation property, simultaneously storing the result (at the same physical location). To access the stored result, excitations are applied until the threshold is reached, the number of excitations required and the calculation base revealing the result. As a practical example, starting in the amorphous phase, we applied excitations of the form in Figure 2c (i.e. 25 × 85 fs pulses = 1 excitation) to perform the summation (7 + 2). Of course the answer is 9 and so the result of the sum should lead to a reflectivity change below the 5% threshold. This was indeed the case; after inputting the first addend (7 excitations) the experimental change in reflectivity was 2.2%; inputting excitations equal to the second addend (2) took the total reflectance change to 4.5%. To access the result of the computation we input further excitations until the threshold is passed; in this case only one further excitation was needed, taking the total experimental reflectivity change to 6.3%, comfortably above the threshold and revealing the correct result of the sum (9 in this case). A microscopic image of the physical mark stored in the phase-change sample as a result of this addition is shown in **Figure** **3** and is just about discernible to the eye. Note that should the result of the sum be greater than the base, the phase-change material is reset to amorphous each time the threshold is exceeded and the number of resets reveals the multiples of the base in the final sum. Re-amorphization is readily achieved in the current arrangement by a single (i.e. 1 × 85 fs) 11.7 mJ cm^−2^ pulse, as also shown in Figure 3.

**Figure 3:**
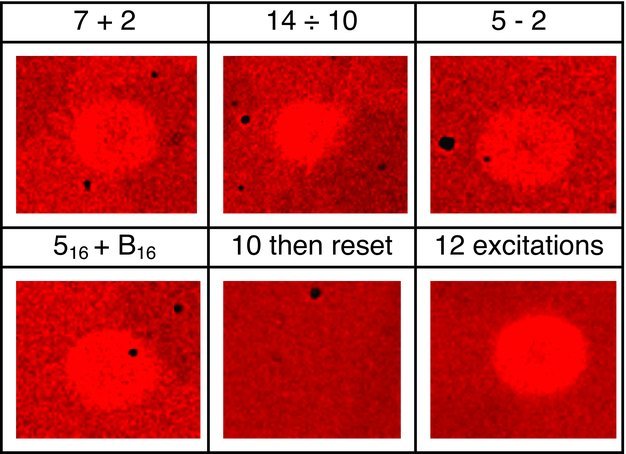
Simultaneous phase-change processing and storage. Microscope images (50 μm × 42 μm in each case) of marks in the Ge_2_Sb_2_Te_5_ sample after the execution of various arithmetic processes. From left to right the first three images show the mark after computing *and* extracting the result for the base-10 computation of 7 + 2, 14÷10 and 5–2. The fourth image shows the mark after computing and extracting the result of the base-16 addition 5_16_+B_16_. For the first two calculations a single excitation comprised a group of 25 × 85 fs optical pulses; for the subtraction calculation a single excitation comprised 50 × 85 fs pulses; for the base-16 calculation a single excitation was 16 × 85 fs pulses. The extraction of the stored result for each of these computations took the measured reflectivity change above the pre-determined threshold value (which was 5%, 5%, 4.5% and 5.4% respectively), so the final marks in each case look very similar. In normal operation the phase-change material is reset to its initial state whenever the threshold is exceeded; in our case this was carried out using a single 11.7 mJ/cm^2^ 85 fs pulse that successfully reset the system to the amorphous phase, as can be seen in the fifth image from the left which shows the result of inputting 10 (25 × 85 fs, 3.61 mJ/cm^2^) excitations followed by a single 11.7 mJ/cm^2^ 85 fs reset pulse. Also shown (far right image) for comparison purposes is the resulting mark after 12 excitations and without resetting; in this case the reflectivity change is ∼11% and the mark is clearly different, even to the eye.

Since multiplication is simply sequential addition, it is clear that this too can be readily implemented using the process described above.

Turning to division, this can be implemented by using the divisor to define the threshold, then applying a number of pulses equal to the dividend (and re-setting each time the threshold is passed). For example 14÷10 is executed by setting the threshold to be passed after 10 input excitations (because this is the divisor, not because we are in base-10) and applying 14 excitations. This would require the system to be re-set once (after the 10^th^ excitation), leaving 4 stored in the phase-change medium; hence the result is 1 remainder 4. We have performed exactly this computation using our phase-change processor. Since we have already set the threshold to occur at 10, which is equal to the divisor in this case, all that remains to perform the division is to input excitations equal in number to the dividend (14), re-setting each time the threshold is reached. Experimentally the measured reflectance change after 10 excitations was 6.3%; this exceeds the threshold so the system was re-set to the amorphous phase, again by a single 85 fs, 11.7 mJ cm^−2^ pulse. A further 4 excitations were then applied, resulting in a negligible change (0.3%) in reflectivity and leaving the remainder (4) of the division calculation stored in the phase-change spot. This remainder is accessed by applying as many subsequent excitations as necessary to once again reach the threshold. This was achieved experimentally with 6 further excitations, which gave a total reflectivity change of 6.0% from the re-set state. Thus the experimental result of the division calculation is as expected, 1 remainder 4, and the final state of the phase-change material upon completion of this division process is also shown in Figure 3.

Finally we turn to subtraction. For conventional computing, division can be done using successive subtraction (e.g. 5÷2 = 2, remainder 1; or 5–2-2 remainder 1); to implement subtraction using a phase-change processor we do the reverse, i.e. use the division algorithm to perform subtraction. For example, to calculate 5–2 we use the minuend (5) to define the threshold, then we input excitations equal to the subtrahend (2); the phase-change material carries out the subtraction and simultaneously stores the result (3 in this case), which is accessed by counting the number of input pulses (3) required to reach threshold. We have re-cast the subtraction (5–2) as a division (5÷2) and carried out our previous division process but this time with the dividend (5) defining the threshold (rather than the divisor). An alternative view of subtraction is as the addition algorithm but with the threshold set by the minuend, rather than by the base. To perform the calculation 5–2 experimentally we first set the threshold to be exceeded after 5 excitations (the minuend in this example). We can do this easily in our system by grouping the basic 85 fs, 3.61 mJ cm^−2^ pulses into excitation units of 50 pulses (i.e. one excitation event is 50 × 85 fs pulses). The typical reflectivity change after 4 such excitations is 3% and that for 5 excitations is 6%, thus a suitable threshold reflectivity change in this case is 4.5%. All that remains to perform the calculation is to input to the system a number of excitations equal to the subtrahend (2), the phase-change material then executes the computation and simultaneously stores the result (3). Experimentally the reflectivity change obtained following the input of the subtrahend (i.e. 2 excitations) was minimal (0.4%) and to extract the result of the calculation a further 3 excitations were required to exceed the threshold, as expected. The total reflectivity change following input of these 3 further excitations was 5.8%, significantly above the threshold, while the reflectivity change after inputting only 2 excitations was well below the threshold. An image of the mark at the end of this subtraction process is also shown in Figure 3. Although not demonstrated here, it is also easy to see that subtractions resulting in a negative difference can be directly implemented using the same approach.

As a further demonstration of arithmetic processing we execute directly a hexadecimal computation, specifically the sum 5_16_+B_16_ (= 10_16_; remember that the basic hexadecimial digits are represented by 0, 1, 2 …..9, A, B, C, D, E, F). For base-16 addition we set the threshold reflectivity change to lie between the 15^th^ and 16^th^ excitations. We do this by combining the basic 85 fs pulses into groups of 16 such that a single excitation event consists of 16 × 85 fs pulses and for which the threshold reflectivity change is 5.4%. The hexadecimal addition is then carried out by inputting 5_16_ excitations (i.e., 5_10_ × 16, 85fs pulses) followed by B_16_ (i.e. 11_10_ × 16, 85 fs pulses). Experimentally this resulted in reflectivity changes of 0.4% and 6.0%, respectively, providing the correct answer of 10_16_; the image of the mark at the end of this process is also shown in Figure 3. A summary of all the above arithmetic computations is given in **Table** **1** and we note that the main source of uncertainty in such computations stems from variations in the power of the probe beam used for our reflectivity measurement and slight changes caused to the optical path when moving the sample–such variations could be significantly reduced in a dedicated system, so providing for reliable computation.

**Table 1:** Summary of experimentally performed arithmetic processes including definition of a single excitation event for each calculation, the threshold reflectivity change, the excitation sequence (numbers in brackets show number of excitations applied to extract stored result) and the experimentally measured reflectivity change at each stage of the computation

Sum	Excitation Event	Threshold (% R)	Excitation Sequence	Experimental (% R)	Answer (experimental)
7 + 2	25 × 85 fs	5	7, 2, (1)	2.2, 4.5, (6.3)	9
14 ÷ 10	25 × 85 fs	5	10, reset, 4, (6)	6.3, reset, 0.3, (6.0)	1 remainder 4
5–2	50 × 85 fs	4.5	2, (3)	0.4, (5.8)	3
5_16_ + B_16_	16 × 85 fs	5.4	5_10_, 11_10_	0.4, 6.0	10_16_

Finally we now turn our attention to the memristive-like properties of phase-change materials. It has already been pointed out that electrical phase-change memory cells are a form of memristor^8^ (a device whose current state is determined by its excitation history) and that memristive devices can be used to implement synaptic-like processing.^7^, ^8^, ^29^, ^30^ Since we have already shown that phase-change devices can be used to implement a basic form of neuron (Figures 1 and 2 associated text), if we can also implement synaptic-like (memristive-like) processing with phase-change materials then it should be feasible to build entire networks of neurons and their associated synapses using phase-change devices and therefore implement biological-inspired (neuromophic) computation/processing. Indeed, our results (Figure 1b and Figure 2) already demonstrate the optical analogue of a memristor (a memory-reflector or “memflector”) in which the optical reflectivity is determined by excitation history. A distinctive feature of electrical memristance is a non-linear relationship between the integrals of current and voltage, which results in various forms of hysteretic current-voltage (*I*–*V*) curves.^4^, ^8^ The optical equivalent of an *I–V* curve is a plot of reflected (*P_R_*) versus incident (*P_I_*) light intensity. In **Figure** **4** we show such a characteristic *P_R_-P_I_* curve (in this case simulated using an analytical model for the temperature calculation, rate-equation model for calculating the fraction of crystallised material and effective medium theory for calculation of optical properties–see Supporting Information) for several cycles of linear up/down incident laser intensity sweeps; here the reflected intensity continuously increases during sweeps, and the *P_R_-P_I_* slope of each sweep picks up from where the last sweep left off, in direct analogy to the electrical case.^7^, ^8^ Also shown inset in Figure 4 is the laser excitation waveform and the calculated fraction of crystallised material during the various cycles; note that the crystallised fraction is dependent on both temperature and time and that relatively little crystallisation occurs during the first “up” ramp but crystallisation continues during the first “down” ramp (and cooling period) and subsequent cycles in accordance with the well-known time-temperature-transformation (TTT) characteristics of Ge_2_Sb_2_Te_5_.^31^ Figure 4 shows clearly that optical and/or electrical forms of phase-change memflector/memristor devices appear feasible, so offering a synaptic-type processing capability to add to the arithmetic and neuron-like processing already demonstrated above. The remarkable properties of phase-change materials may therefore in time lead to some truly remarkable applications.

**Figure 4:**
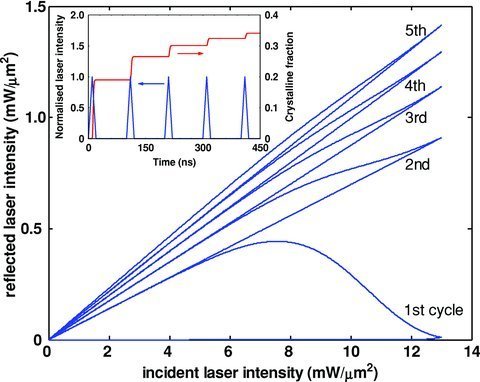
The memflector; optical analogue of the memristor. A plot of the (simulated) normalised reflected light intensity (*P_R_* × ΔR/R_a_) versus incident (*P_I_*) light intensity for the optical analogue of the memristor (the *memflector*) as a function of the number of linear up/down sweeps of the incident laser intensity (this is equivalent to memristor *I–V* curves). Maximum incident intensity was 13 mW/μm^2^ and the up/down ramp time was 20 ns in total (10 ns up and 10 ns down). Also shown inset is the incident laser waveform and resulting fraction of crystallised material.

## Supplementary material

Detailed facts of importance to specialist readers are published as ”Supporting Information”. Such documents are peer-reviewed, but not copy-edited or typeset. They are made available as submitted by the authors.
