# Supplementary material for: Arithmetic and Biologically-Inspired Computing Using Phase-Change Materials
Source: Adv Mater. 2011 Jun 22;23(30):3408–13. doi: 10.1002/adma.201101060 (PMC3715110; doi:10.1002/adma.201101060)
Supplement: Supplementary file 1 [file adma0023-3408-SD1.pdf]

Copyright WILEY-VCH Verlag GmbH & Co. KGaA, 69469 Weinheim, Germany, 2011.

ADVANCED MATERIALS

Supporting Information

for *Adv. Mater.*, DOI: 10.1002/adma.**201101060**

Arithmetic and Biologically-Inspired Computing Using Phase-Change Materials

C. David Wright, Yanwei Liu, Krisztian I Kohary, Mustafa M Aziz, and Robert J Hicken*

Supporting Information

Arithmetic and Biologically-Inspired Computing Using Phase-Change Materials

C. David Wright, Yanwei Liu, Krisztian I Kohary, Mustafa M Aziz and Robert J Hicken*

[*] Prof. C. David Wright Corresponding-Author
University of Exeter
Exeter EX4 4QF, England
E-mail: (david.wright@exeter.ac.uk))
Dr. Y. Liu, Dr K.I.Kohary, Dr. M.M. Aziz and Prof. R.J.Hicken
University of Exeter
EX4 4QF, England

The rate-equation model. The basis of the rate-equation approach is determination of the distribution function, *Z(n, t)*, representing the density of crystal clusters of the size *n* (monomers) at the time *t*. Changes in the crystal cluster sizes occur when monomers are attached to or detached from existing clusters. In all the simulations presented here a discrete version of the rate-equation has been used for which the temporal evolution of the cluster density *Z* is given by[S1]

$$\frac{\partial Z(n,t)}{\partial t} = f(n-1,t)Z(n-1,t) + d(n+1,t)Z(n+1,t) - f(n,t)Z(n,t) - d(n,t)Z(n,t) \qquad (1)$$

where *f(n, t)* and *d(n, t)* are, respectively, the rates of attachment to and detachment from a crystal cluster of size n units (monomers) and depend strongly on temperature (primarily via (i) Boltzmann factors defined in terms of Gibbs free energy differences between cluster sizes *n* and *n+1* and (ii) a jump frequency at the crystal/amorphous interface). As an example of the output of the evolution of cluster size distribution as predicted via the rate-equation model we show in Fig. S1 the population density of clusters (of sizes *n*=1 to *n*=40) at the end of the 1st, 2nd, 5th and 10th temperature pulses for the 10 ns, 700 K case of Fig. 1 in the main paper. Note that the crystal fraction at any time *t* is found by integrating the cluster population distribution function *Z(n,t)* for all cluster sizes above the critical cluster size. This approach

yielded the simulated crystal fraction and, via the effective medium approximation, the associated optical reflectivity and electrical conductivity values for the results of Fig. 1b and Fig. 2b and 2c of the main paper. Key parameters used in such simulations are the enthalpy *H* (here taken to be 1121 Jcm^{-3} and taken from[S2]), the interfacial surface energy (0.1 Jm^{-2}, from[S1]), activation energy (2 eV from[S1] and the viscosity (1.95 x 10^{-14} Pa.s, also from[S1]. For the results of Fig. 1b and Fig. 2b and 2c we assumed heterogeneous nucleation (simulated via the spherical cap model[S3] with $\theta=85^0$), since it is well known[S2,S4] that in optical storage media nucleation occurs preferentially at interfaces.

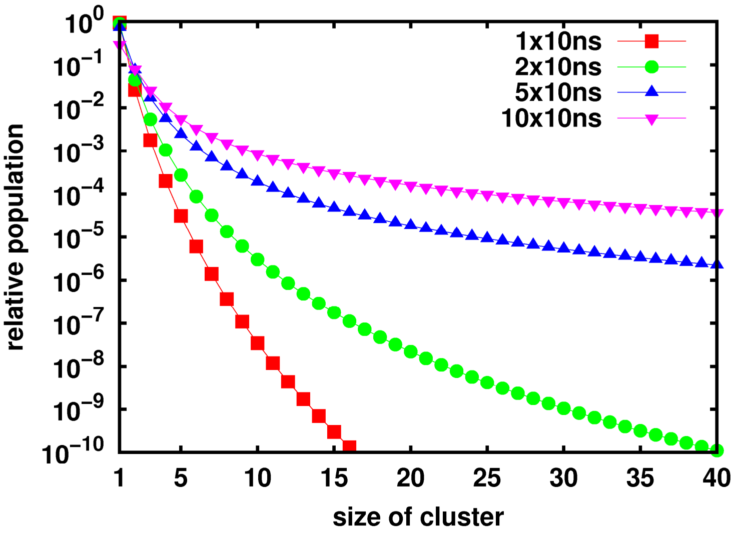

Figure S1 Population density of crystal cluster sizes after 1, 2, 5 and 10 temperature pulses for the 10 ns, 700 K pulse case of Fig 1b in main paper

Effective medium approximation. Optical and electrical properties of a two component mixture of amorphous and crystalline phases were calculated using effective medium theory. For our case of spherical crystallites growing in an amorphous matrix, the dielectric function is given by[S5]

$$\varepsilon(f,\varepsilon_a,\varepsilon_c)=\frac{1}{4}\left(2\varepsilon_p-\varepsilon_p^*+\sqrt{\left(2\varepsilon_p-\varepsilon_p^*\right)^2+8\varepsilon_a\varepsilon_c}\right) \quad (2)$$

where f is the crystal fraction, ε_a and ε_c are the dielectric constant of the (fully) amorphous and crystalline phases and $\varepsilon_p = (1-f)\varepsilon_a + f\varepsilon_c$ and $\varepsilon_p^* = (1-f)\varepsilon_c + f\varepsilon_a$. To calculate optical reflectivity in Fig. 1b, Fig. 2b, Fig. 2c and Fig. 4 of the main paper we used refractive indices (at 632 nm, the wavelength of the probe beam) for (fully) amorphous and crystalline phases of n_a = 4.068−i2.060 for the amorphous state and n_c =3.871−i4.266, taken from[S5], and assumed, for simplicity, an air/$Ge_2Sb_2Te_5$ interface and normal incidence. As shown previously[S5], the relationship between crystal fraction and optical reflectivity is essentially linear. To calculate electrical conductivity we simply replace the dielectric function in the above equations by the appropriate electrical conductivity σ, here using values[S6] for σ_a and σ_c of 0.4 $\Omega^{-1}m^{-1}$ and 3250 $\Omega^{-1}m^{-1}$.

Temperature calculations. The one-dimensional (consistent with the large diameter of the laser spot compared to the sample thickness), parabolic, heat conduction equation:

$$\frac{1}{\alpha}\frac{\partial T}{\partial t} = \frac{\partial^2 T}{\partial x^2} + \frac{g(x,t)}{k} \quad (3)$$

was solved for the temperature T in two layer structure that models the experimental sample and consisted of a phase-change layer on top of a semi-infinite underlayer. k and α in (3) are the thermal conductivity and diffusivity of the layers respectively, and g is the laser heating source power density active only in the phase-change layer. Integration of the heat equation subject to thermal insulating boundary at the top of the phase-change layer, and room temperature T_o at the bottom of the semi-infinite underlayer yields the solution:

$$T_p(x,t) = T_o + \frac{\alpha_p}{k_p}\int_{\tau=0}^{t} d\tau \int_{x'=0}^{x_p} g(x',\tau)\cdot G_p(x,x';t-\tau)dx' \quad (4)$$

for the temperature in the phase-change layer in terms of the Green function G_p solution of the equivalent homogenous boundary value problem, where the integration is through the

thickness (x_p = 20 nm) of the phase-change layer. The laser absorption through the thickness (x dimension) of the phase-change layer was modelled as an exponential internal energy density distribution described by:

$$g(x,t) = q_o A(1-R)\exp(-Ax)\,p(t) \qquad (5)$$

where q_o is the peak power density of the laser source, $A = 4\pi\kappa/\lambda$ is the absorption depth, κ is the extinction coefficient of the amorphous starting phase (taken here to be 1.5 from[S7]) at wavelength λ = 800 nm for the pump laser beam, R is the reflectivity of the amorphous phase (37% as measured experimentally in this work) and $p(t)$ is the temporal profile of the laser source. Other key parameters used were: thermal conductivity k_p = 0.23 W/(mK) and α_p = 179x10^{-9} m^2/s for the amorphous starting phase. The effects of the thermal anisotropies and release of latent heat during the phase transition were assumed negligible for the class of phase-change material considered in this work, a reasonable approximation as found previously[S8,S9].

For the femto-second laser heating simulations, a Dirac delta temporal profile for the heat source was used in (3) with $p(t) = t_o\delta(t)$ where t_o is the pulse width and set to 85 fs to match the experimental laser pulse duration. The repetition rate of the laser pulses was 100 kHz, in line with experiment, and ensures that the steady-state room temperature is reached before the next pulse is applied without any heat energy accumulation from consecutive laser pulses. Equation (4) was integrated exactly and the leading terms of the solution were taken as they dominate at short time scales. Figure S2 shows the calculated temperatures at the top and middle points of the phase-change layer from the analytical solution using the experimental peak laser energy density or fluence (=$q_o t_o$) of 3.61 mJ/cm^2. We compared the predicted temperature distribution according to this solution to a full finite-element numerical solution that takes into account the influences of a capping layer and the finite thickness underlayer;

the analytical solution was found to be a good match (particularly at short time-scales, i.e. < 1 ns, where the numerical solution tends to the Green function solution) and so was used directly in the rate-equation simulation to estimate the fraction of crystallised material for Fig. 2 of the main paper. We note that the equilibrium, parabolic heat conduction model (considered here for simplicity) does not account for the transfer of energy between electrons and phonons during the transient heating process at short time scales, which will be considered in a future publication.

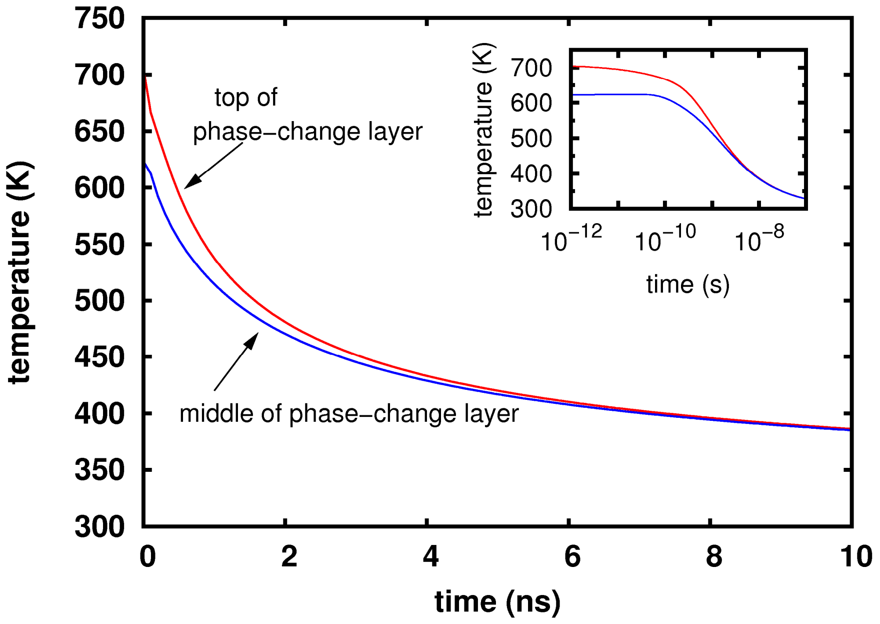

Figure S2 Simulated temperature at the top (solid line) and middle (dashed line) of the 20 nm thick phase-change layer as a function of time for a single 85 fs, 3.61 mJ/cm^2 pulse. Also shown (inset) is the same result on a log time scale. This temperature profile was used to calculate the theoretical results shown in the main paper in Fig. 2b and Fig. 2c.

For the memflector simulations, the heat distribution in the phase-change layer was determined analytically from (4) using a triangular up/down ramp of laser power with temporal profile:

$$p(t) = \begin{cases} \dfrac{t}{t_p}, & 0 \le t < t_p \\ \dfrac{-t}{t_p} + 2, & t_p \le t \le 2t_p \end{cases} \qquad (6)$$

where t_p is the peak time of the triangular pulse which was taken to be 10 ns and using a peak laser power density q_o = 13 mW/µm^2 (13 GW/m^2) that is sufficient to crystallise the amorphous phase-change layer after several cycles. Equation (4) was integrated using the laser pulse profile in (6) yielding an exact solution for the transient temperature distribution at the top of the phase-change layer, as shown in Fig S3. The crystalline fraction was then calculated from the amorphous starting phase using the rate equation model and the corresponding change in refractive index and hence reflectivity for each applied pulse was calculated using the crystalline fraction and complex dielectric constant from (2).

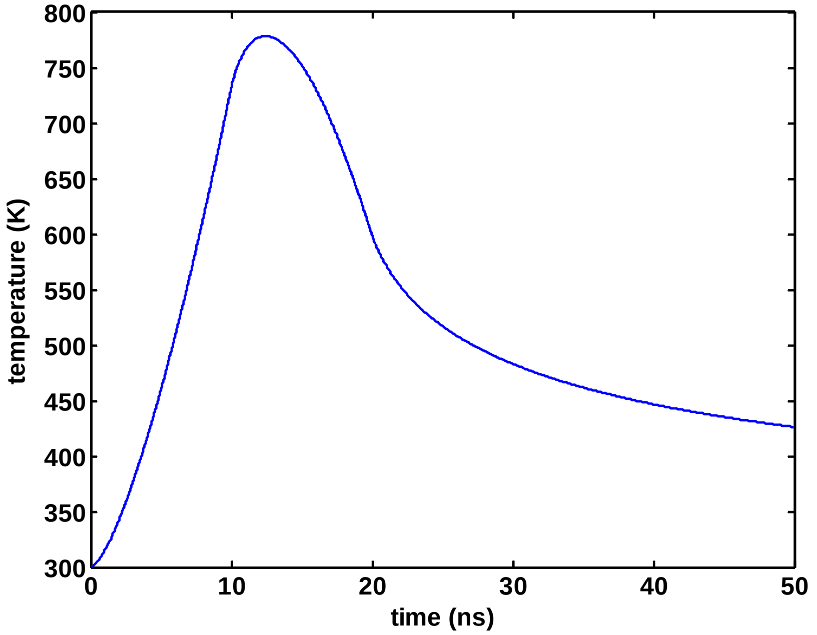

Figure S3 Calculated temperature distribution at the top of the phase-change layer for triangular up-down incident laser power ramp of 20 ns duration (10 ns up and 10 ns down) and 13 mW/µm^2 peak intensity.

Supporting References

[S1] S. Senkader and C.D. Wright, *Appl. Phys*. **2004**, *95*, 504

[S2] B. Hyot et al. *J. Magn. Soc. Jpn.* **2001**, *25*, 414

[S3] J. W. Christian, *The Theory of Transformations in Metals and Alloys* ~Pergamon, Oxford, (1975)

[S4] N. Yamada et al. *Jpn. J. Appl. Phys.***1998**, *37*, 2104

[S5] D-H. Kim D-H. et al. *J. Appl. Phys*. **2005**, *97***,** 083538

[S6] C. D. Wright, K. Blyuss and P. Ashwin, *Appl. Phys. Lett.* **2007**, *90*, 063113

[S7] A. Chalbi et al. *J. Magn. Mag. Mater.* **2002**, *249,* 509

[S8] M.R. Belmont et al. *J. Appl. Phys.***2008**,*104,* 044901

[S9]. M.M. Aziz et al. *J. Appl. Phys*. **2008**,*104*, 104912
